# Effect of vitamin D on anterior cruciate ligament injury rates and post‐reconstruction function—A systematic review

**DOI:** 10.1002/jeo2.70224

**Published:** 2025-04-01

**Authors:** Andrea Pasquini, Radu Prejbeanu, Octav Russu, Riccardo D'Ambrosi, Cosmin Ioan Faur, Giulio Maria Marcheggiani Muccioli, Mihail Lazar Mioc

**Affiliations:** ^1^ Department of Orthopaedics and Traumatology “Pius Brinzeu” Emergency Clinical County Hospital Timisoara Romania; ^2^ Center for Modeling Biological Systems and Data Analysis, “Victor Babes” University of Medicine and Pharmacy Timisoara Romania; ^3^ Department XV‐Discipline of Orthopedics‐Traumatology “Victor Babes” University of Medicine and Pharmacy Timisoara Romania; ^4^ UMFST G.E. Palade Tg. Mures Târgu Mureș Romania; ^5^ IRCCS Istituto Ortopedico Galeazzi Milan Italy; ^6^ Dipartimento di Scienze Biomediche per la Salute Università degli Studi di Milano Milan Italy; ^7^ II Orthopaedic and Traumatologic Clinic, IRCCS Istituto Ortopedico Rizzoli Bologna Italy; ^8^ Dipartimento di Scienze Biomediche e Neuromotorie DIBINEM University of Bologna Bologna Italy

**Keywords:** anterior cruciate ligament (ACL), anterior cruciate ligament injury, anterior cruciate ligament reconstruction (ACLR), functional outcome, muscle strength, serum vitamin D level, vitamin D

## Abstract

**Purpose:**

This systematic review aimed to investigate the association between serum vitamin D levels and key outcomes following anterior cruciate ligament reconstruction (ACLR), including ACL injury risk, postoperative muscle recovery and post‐reconstruction functional outcomes.

**Methods:**

We conducted a comprehensive search across five databases (Cochrane Library, EMBASE, MEDLINE, Scopus and Web of Science) until July 2024. Studies were selected based on specific inclusion criteria, such as studies evaluating the relationship between vitamin D and injury risk, ACLR outcomes and muscle strength. Risk of bias was assessed using the MINORS tool, and results were synthesized narratively due to study heterogeneity.

**Results:**

Five studies with 656,243 participants met the inclusion criteria. Most studies reported that low vitamin D levels were associated with a significantly increased risk of ACL injuries and poorer postoperative muscle strength recovery. However, evidence regarding bone health and functional outcomes was inconsistent. No meta‐analysis was conducted due to the variability of study designs and outcomes, but qualitative synthesis indicated a potential protective role of vitamin D in ACL recovery.

**Conclusions:**

Vitamin D deficiency appears to increase the risk of ACL injury and impede muscle recovery post‐surgery. However, limitations include a high risk of bias and inconsistent evidence on functional outcomes, underscoring the need for further research. The review was registered in PROSPERO (ID: CRD42024584483).

**Level of Evidence:**

Level III, systematic review.

**Abbreviations**
ACL: anterior cruciate ligament
ACLR: anterior cruciate ligament reconstruction
BMD: bone mineral density
CCLR: cranial cruciate ligament rupture
CSA: cross‐sectional area
LoE: level of evidence
MINORS: Methodological Index for Non‐Randomized Studies
MVIC: maximal voluntary isometric contraction
PRISMA: Preferred Reporting Items for Systematic Reviews and Meta‐analyses
RCT: randomized controlled trial
RTD: rate of torque development

## INTRODUCTION

Anterior cruciate ligament (ACL) injuries are among the most prevalent and debilitating injuries in sports medicine. They frequently require surgical reconstruction to restore knee stability and function. Despite advancements in surgical techniques and rehabilitation protocols, the success of ACL reconstruction (ACLR) varies widely among patients. Emerging evidence suggests that nutritional factors, particularly vitamin D status, play a significant role in influencing the outcomes of ACL reconstruction.

Vitamin D is a fat‐soluble vitamin essential for calcium homoeostasis and bone metabolism. It has widespread biological effects, including roles in muscle function, immune modulation and inflammation regulation. Deficiency in vitamin D is a global health concern, affecting up to 77% of the population, with athletes being particularly susceptible due to high physical demands and potential limited sun exposure. Optimal levels of vitamin D are critical for musculoskeletal health, and insufficiency has been linked to muscle weakness, impaired neuromuscular function and increased injury risk [13, 15, 19, 21].

The functions of vitamin D in relation to bone health, calcium homoeostasis, and the risk of stress fractures have been substantiated by both biomolecular and clinical research. Clinically deficient levels of vitamin D have been associated with a markedly increased occurrence of stress fractures in athletes and physically active individuals, in contrast to those with sufficient vitamin D levels. Both high‐energy and low‐energy traumatic fractures are associated with vitamin D deficiency. Considering the importance of vitamin D for skeletal health, it is imperative to identify and address deficiencies in order to optimize patient outcomes [36].

A deficiency in vitamin D may lead to impairments in muscle function and performance, thereby impacting the rehabilitation process. Furthermore, research has elucidated the involvement of vitamin D in the mechanisms of pain pathways. In a retrospective analysis of orthopaedic surgical cases, patients with orthopaedic trauma exhibited a 66% incidence of low vitamin D levels, while those engaged in athletics demonstrated a 52% incidence of the same deficiency. In a study involving 577 consecutive patients undergoing elective foot and ankle surgery, it was observed that only one in six patients exhibited normal vitamin D levels, while one in five patients demonstrated gross deficiency in this vitamin [3].

The relationship between vitamin D and outcomes subsequent to ACL reconstruction (ACLR) remains relatively under‐researched. It has been noted that there is a temporary reduction in circulating levels of 25‐hydroxyvitamin D (25(OH)D) immediately following the acute trauma associated with ACLR. Furthermore, diminished circulating levels of 25(OH)D appear to exacerbate the strength deficits in the affected limb subsequent to ACLR. More recently, primary ACL tears, revision ACLR, and skeletal muscle atrophy after ACLR have been found to increase with reduced serum 25(OH)D concentrations. Although these findings suggest a correlation between low vitamin D levels and risk factors associated with knee osteoarthritis (such as ACL injuries and skeletal muscle atrophy and weakness), it remains unclear whether low or deficient serum 25‐hydroxyvitamin D (25(OH)D) is associated with the incidence of knee osteoarthritis and a reduced disease‐free survival following ACLR.

Gupta et al. reported that vitamin D deficiency in athletes did not significantly alter functional outcomes following ACL reconstruction, although the study acknowledged limitations such as not measuring postoperative vitamin D levels, which could provide more comprehensive insights into the recovery process [5, 12].

In contrast, a study by Qiu et al. highlighted significant correlations between serum vitamin D levels and quadriceps neuromuscular function in ACL‐injured patients. This study found that insufficient serum vitamin D concentrations were associated with decreased maximal voluntary isometric contraction (MVIC), slower torque development rate (RTD), and increased quadriceps inhibition, particularly in the uninjured limb. These neuromuscular deficits could impede effective rehabilitation and increase re‐injury risk [4, 26].

Furthermore, Wen et al. demonstrated that vitamin D status is closely linked to skeletal muscle mass and function post‐ACL reconstruction. Their findings suggest that adequate vitamin D levels may mitigate muscle atrophy and enhance recovery, emphasizing the importance of monitoring and addressing vitamin D insufficiency in patients undergoing ACL surgery [20, 22, 36].

Previous research highlights the role of imaging techniques in evaluating surgical outcomes in ACL reconstruction, with magnetic resonance imaging and computed tomography proving valuable for assessing graft positioning and tunnel accuracy. These studies also highlight the importance of bone healing processes in recovery, suggesting that mechanical and biological factors play a critical role in successful postoperative outcomes [34, 35].

Nonetheless, there is no definitive data indicating that vitamin D insufficiency negatively impacts the outcomes of ACL surgery.

This systematic review will evaluate whether maintaining sufficient vitamin D levels can improve surgical outcomes, enhance rehabilitation efficacy and reduce the risk of complications. By collating and critically appraising the available data, we seek to provide clearer clinical practice guidance and identify future research directions.

We hypothesize that elevated serum vitamin D levels in patients will be associated with improved functional outcomes following ACL reconstruction.

This systematic review aimed to investigate the association between serum vitamin D levels and key outcomes following ACLR, including ACL injury risk, postoperative muscle recovery, and post‐reconstruction functional outcomes.

## MATERIALS AND METHODS

### Search strategy

To develop a focused and effective search strategy, we conducted our research question using the PICO framework:

### PICO framework

− P (Population): Adult patients who underwent ACL reconstruction.
− I (Intervention): Adequate vitamin D levels (or higher vitamin D levels, depending on how the authors categorized their groups).
− C (Comparison): Inadequate or lower vitamin D levels.
− O (Outcome): functional recovery, injury rates and revision incidence, biomarkers, bone and muscle health, patient‐reported outcomes.

This framework guided the development of a search strategy, incorporating specific inclusion and exclusion criteria to systematically identify relevant studies. All study designs, including cross‐sectional, case–control, cohort and randomized controlled trials (RCTs), were considered. The focus was on adult patients undergoing ACL reconstruction with reported vitamin D levels, whether observational or involving interventions.

A literature search was conducted on five databases (Cochrane Library, EMBASE, MEDLINE (PubMed), Scopus and Web of Science), with no time limitation and with the last update being July 2024. Preferred Reporting Items for Systematic Reviews and Meta‐Analysis statement (PRISMA) was used as a guideline to identify all relevant studies [23]. Additionally, the references cited in the identified studies were examined to find any further studies that met the inclusion criteria.

The search combined “Anterior Cruciate Ligament Reconstruction” AND “Vitamin D” descriptors. See Appendix S1 for the extended form.

This study was registered on the International Prospective Register of Systematic Review (PROSPERO registration number: 2024 CRD42024584483) [24]. Ethical committee approval was not required for this study.

### Eligibility criteria

Inclusion criteria were as follows: (1) studies evaluating the relationship between ACLR and vitamin D, (2) studies of Levels I to IV evidence, RCTs, controlled (nonrandomised) clinical trials (CCTs), prospective and retrospective comparative cohort studies, case‒control studies and case series, (3) use of human subjects, (4) articles written in English and (5) full text available. Exclusion criteria were: (1) papers not published in English, (2) reviews and (3) laboratory studies.

### Participants

Studies were conducted on skeletally mature patients who had ACL injury or undergone ACL reconstructions and where serum level of vitamin D was evaluated. Patients undergoing concomitant procedures or revision surgeries were not excluded if the main surgery was ACL reconstruction.

### Study selection and data extraction

Article selection, through screening at every level and for each study, was performed by two authors (MM and AP) in a blinded way in order to eliminate inter‐observer bias. The abstract and title of all identified studies were initially reviewed, followed by an in‐depth analysis of each full text.

Two independent reviewers (MM and AP) collected all relevant data using a predefined spreadsheet in Microsoft Excel, 2019 version (Microsoft Corporation).

### Data collection process

The data were extracted from the selected articles by the first two authors using a computerized tool created with Microsoft Access (Version 2010, Microsoft Corp). Each article was validated again by the first author before analysis. For each study, data regarding the patients were extracted.

### Level of evidence (LoE), quality assessment and risk of bias

Two reviewers (OR and CF) independently assessed the quality of each included study, without blinding, using the scoring protocol developed by the Oxford Centre for Evidence‐Based Medicine to determine LoEs (Table 1) [25].

**Table 1:** Study characteristics.

					N (Male)			N (Female)			Age (years)		
Authors	Year	Design	LoE	MINORS score	GR 1	GR 2	GR 3/CTRL	GR 1	GR 2	GR 3/CTRL	GR 1	GR 2	GR 3/CTRL
Qiu et al. [7]	2024	Cross‐sectional	II	9/16	14	N/A	N/A	4	N/A	N/A	28.0 median (21.8, 33.3)	N/A	N/A
Albright et al. [18]	2023	Retrospective database	III	17/24	112, 180	112, 180	N/A	215, 831	215, 831	N/A	41.9 ± 12.6	41.9 ± 12.6	N/A
Wen et al. [9]	2023	Cohort, Prospective	II	20/24	8	N/A	N/A	13	N/A	N/A	17.8 ± 3.2	N/A	N/A
Gupta et al. [5]	2021	Cohort, Prospective	II	19/24	46	48	48	5	3	3	24.12 ± 2.12	25.24 ± 3.20	24.74 ± 2.86
Barker et al. [6]	2011	Cohort, Retrospective	III	17/24	9	9	11	0	0	0	35 ± 2	29 ± 3	31 ± 1

Abbreviations: CTRL, control group; GR, group; LoE, Level of Evidence; MINORS, Methodological Index for Non‐Randomized Studies; N/A, not applicable.

For study quality assessment, the MINORS tool is appropriate for non‐comparative studies to identify common sources of bias, such as lack of prospective data collection, non‐comparability of groups, and incomplete follow‐up [29]. According to these criteria, non‐comparative studies can achieve a maximum score of 16, while comparative studies can score up to 24. For non‐comparative studies, classification was based on a previous systematic review as follows: 0–6 low quality, 7–12 moderate quality and scores ≥13 high quality. Comparative studies were categorized as: 0–10 low quality, 11–16 moderate quality and ≥17 high quality.

For RCTs, the Risk of Bias (RoB2.0) tool would be suitable to evaluate study quality, focusing on randomization, allocation concealment, blinding and outcome reporting [30].

Potential impacts of identified biases on the review findings will be discussed in the results and limitations sections [29].

Results for LoE and risk of bias were confronted and disputes were solved by a third independent author (RD).

### Data analysis

Due to a limited number of studies eligible for inclusion, a statistical comparison of outcomes was not performed.

## RESULTS

### Search strategy and literature selection

**Figure 1:**
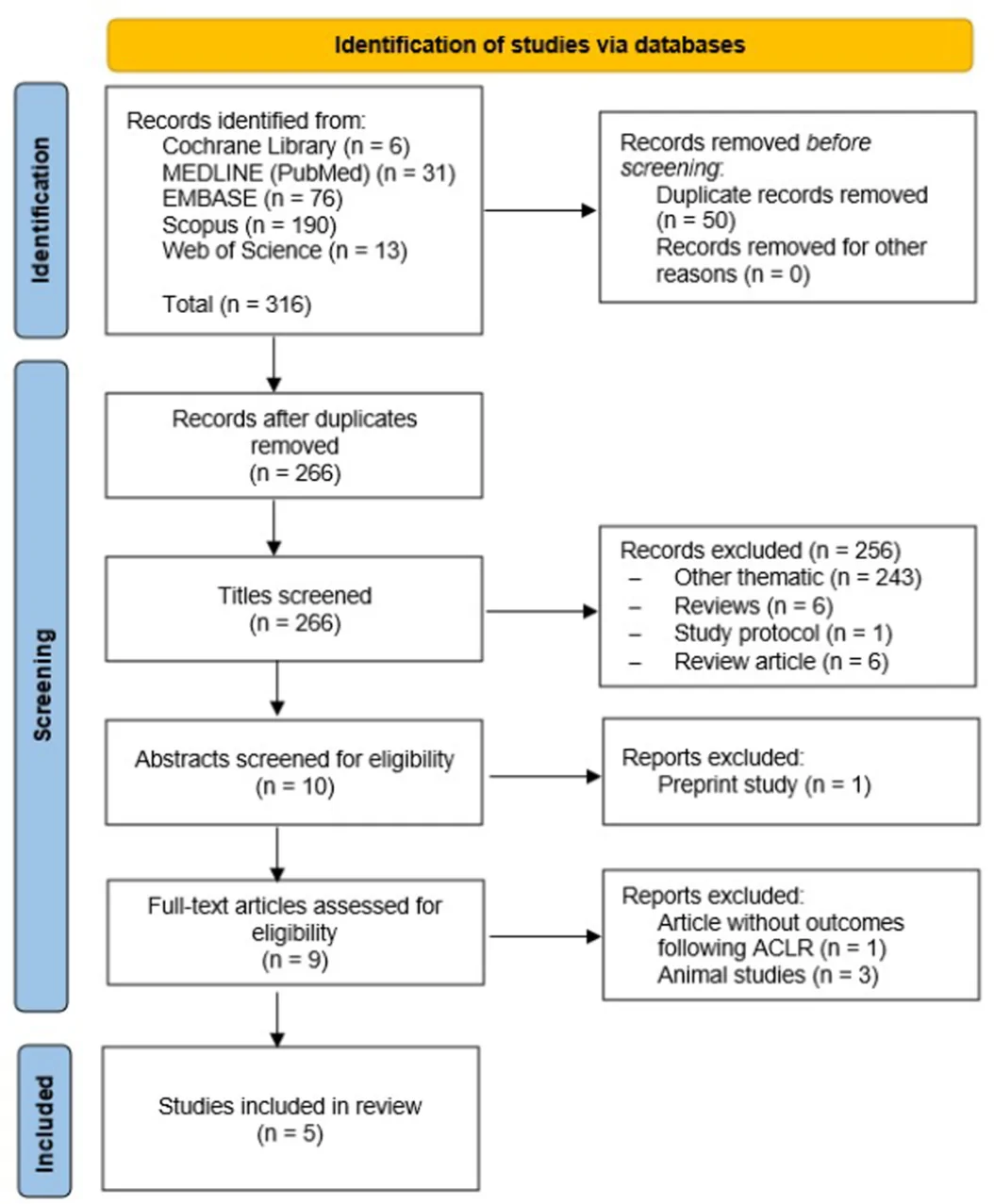
Flowchart of the literature search. ACLR, anterior cruciate ligament reconstruction.

Initially, the search identified 316 articles, 50 of which were duplicates. Of the remaining 266 articles, 256 were eliminated because they did not fit the study's inclusion criteria. Of the remaining 10 articles, one was excluded due to its preprint status, another for lacking outcomes related to ACL reconstruction, and three others were excluded as they involved animal studies. No articles were identified through the reference lists. After the exclusion process, five articles were included in this systematic review (Figure 1).

### Study characteristics

The included articles consisted of two prospective cohort studies (Level II evidence), one retrospective cohort study (Level III evidence), one retrospective database study (Level III evidence) and one cross‐sectional study (Level II evidence). There was no disagreement between the two reviewers in the selection of articles. Cohen's kappa showed excellent agreement between the two readers (0.99). The ICC for the reliability of article selection was 0.99.

In total, there were 656,243 participants, of which 224,553 were me, and 431,690 were women (Table 1). The high number of patients is due to a database study (Albright et al.), which represents approximately 99% of the total patients in our study; nevertheless, the population in the study is highly representative as a model for general population, as it comprises patients within a wide age range (10–59 years), both sexes and with heterogeneous comorbidities.

### Quality assessment

**Figure 2:**
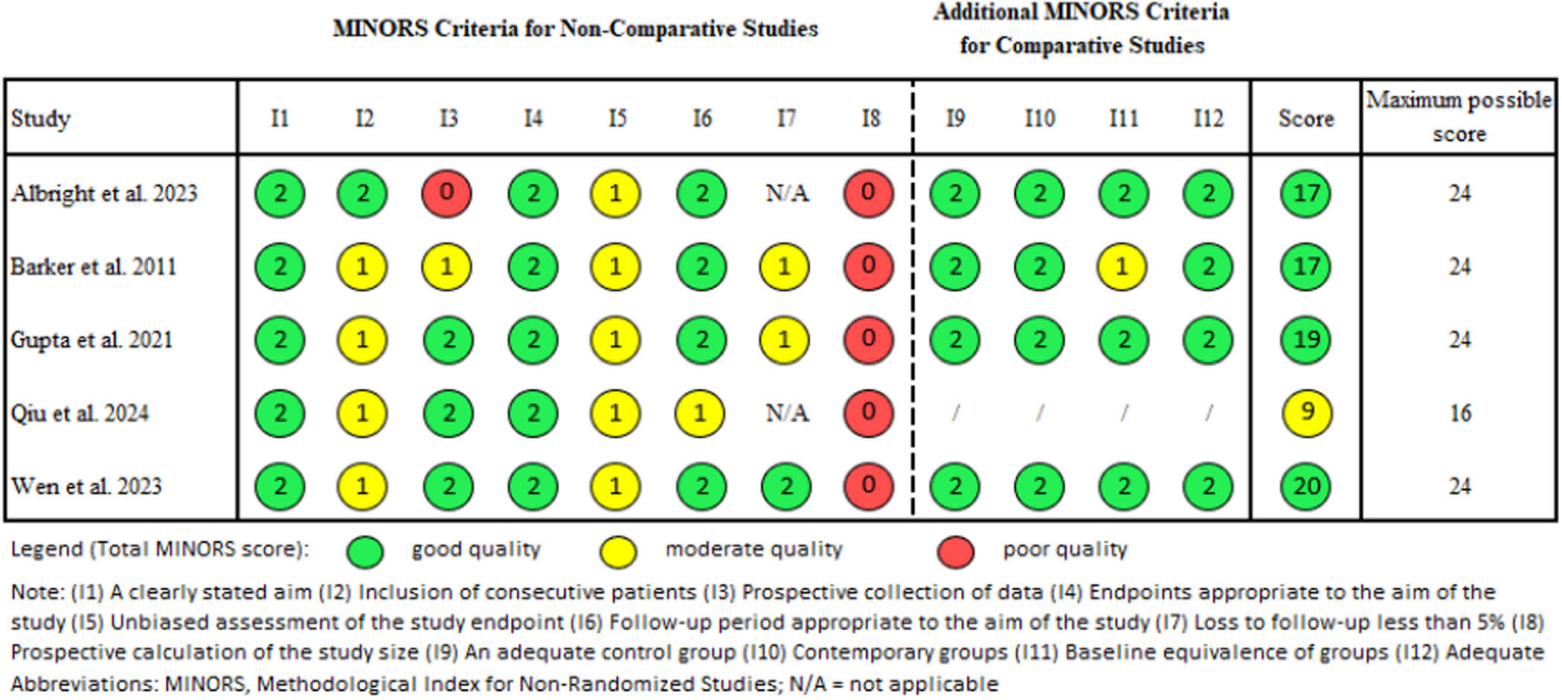
Quality assessment criteria for non‐randomized surgical studies. MINORS, Methodological Index for Non‐Randomized Studies.

All the comparative studies included scored in the high‐quality range (≥17). The only non‐comparative study included was considered to be of moderate quality (7–12 score range) (Figure 2).

### Outcomes

This systematic review synthesized findings from five studies that investigated the impact of vitamin D on various outcomes related to ACL injury and reconstruction (Table 2).

**Table 2:** Outcomes of included studies.

Study	Outcomes evaluated
Qiu et al. 2024	Speed of rapid contraction (RTD)
	Quadriceps strength (MVIC)
	Quadriceps inhibition (CAR)
	Serum vitamin D concentration [25(OH)D]
Albright et al. 2023	Incidence of ACL tear at 1 year
	Incidence of ACL tear at 2 years
	Rate of primary reconstruction
	Rate of revision reconstruction
Wen et al. 2023	Circulating biomarkers [1,25(OH)_2_D]
	Quadriceps fibre CSA
	BMD
	Quadriceps strength (MVIC)
	Speed of rapid contraction (RTD)
Gupta et al. 2021	WOMAC Score
	Lysholm Score
	Tegner activity score
Barker et al. 2011	Plasma cytokines concentrations
	Single‐leg peak isometric forces

Abbreviations: ACL, anterior cruciate ligament; BMD, bone mineral density; CAR, central activation ratio; CSA, cross‐sectional area; ELISA, enzyme‐linked immunosorbent assay; MVIC, maxim voluntary isometric contraction; RTD, rate of torque development; WOMAC, Western Ontario and McMaster Universities Arthritis Index.

The outcomes were categorized into three main areas: ACL injury risk, postoperative muscle strength recovery, and functional outcomes, including neuromuscular function and bone health.

### ACL injury risk

Albright et al. identified a significant relationship between vitamin D deficiency and an increased risk of ACL injuries [2]. In a retrospective cohort study of over 328,000 patients, individuals with low serum 25‐hydroxyvitamin D levels (<30 ng/mL) exhibited a notably higher incidence of ACL tears—115.2 per 100,000 person‐years—compared to 61.0 per 100,000 person‐years in patients with sufficient vitamin D levels. The adjusted odds ratio indicated that patients with vitamin D deficiency were 81% more likely to sustain an ACL tear within 2 years than those with adequate levels. These results underscore the role of vitamin D as a potential modifiable risk factor for ACL injury, particularly among high‐risk populations such as athletes. This finding suggests that screening for and correcting vitamin D deficiency may serve as a preventive measure against ACL tears, providing an avenue for further research into the protective role of vitamin D supplementation in musculoskeletal injury prevention.

### Postoperative muscle strength recovery

Three studies examined the impact of vitamin D levels on muscle strength recovery following ACL reconstruction. Barker et al. investigated 29 patients who underwent ACL surgery and found that those with lower plasma 25‐hydroxyvitamin D concentrations (<30 ng/mL) exhibited significantly reduced improvements in muscle strength recovery three months post‐surgery compared to those with higher vitamin D levels. Specifically, the increase in peak isometric force in the injured limb was nearly sixfold greater in participants with sufficient vitamin D levels. Similarly, Wen et al., in a prospective cohort study of 21 participants, reported that patients with low vitamin D levels experienced significantly greater reductions in quadriceps muscle fibre cross‐sectional area (CSA) at 1 week and 4 months post‐ACLR. This study did not find a significant impact of vitamin D on bone mineral density (BMD) loss, but the muscle preservation aspect was clear. Additionally, Qiu et al., in a cross‐sectional study of 18 ACL‐injured patients, observed that vitamin D insufficiency correlated with impaired quadriceps neuromuscular function, particularly in the uninjured limb. Higher vitamin D levels were associated with greater muscle strength and better neuromuscular control, which is crucial for preventing further injury and enhancing recovery.

### Functional outcomes

Gupta et al. studied 153 athletes who underwent ACL reconstruction and grouped them by their preoperative vitamin D levels: Group 1 (<20 ng/mL), Group 2 (20–30 ng/mL) and Group 3 (>30 ng/mL). The study found no significant differences in functional outcomes across these groups at 2 years post‐surgery, including Lysholm and WOMAC scores. However, those with adequate vitamin D levels had slightly better Tegner activity scores, suggesting a potential benefit in return to sport.

Qiu et al. reported a significant positive correlation between vitamin D levels and quadriceps neuromuscular function, particularly in the uninjured limb. This finding is important as it suggests that maintaining adequate vitamin D levels could help prevent contralateral ACL injuries and improve overall knee stability.

### Bone health and skeletal muscle loss

Wen et al. explored the relationship between vitamin D status and skeletal muscle loss following ACL reconstruction. Their study found that lower vitamin D levels were associated with greater reductions in quadriceps muscle fibre CSA post‐ACLR. However, there was no significant association between vitamin D levels and BMD loss in the proximal tibia and distal femur. This suggests that while vitamin D is crucial for muscle preservation during recovery, its role in maintaining bone density after ACLR is less clear.

## DISCUSSION

This systematic review highlights the potential role of vitamin D in ACL injury risk, postoperative muscle recovery, functional outcomes and bone health following ACLR. Our findings suggest that low vitamin D levels may be associated with an increased risk of ACL injury, possibly due to its role in muscle function and inflammation regulation. Additionally, patients with vitamin D deficiency show impaired muscle recovery post‐ACLR, with evidence pointing towards its involvement in muscle protein synthesis and neuromuscular function. While vitamin D may contribute to functional recovery, its direct influence on RTS outcomes remains inconclusive. Similarly, its role in bone health after ACLR is not well‐established, with conflicting evidence regarding its impact on BMD. These findings highlight the potential benefits of vitamin D optimization in ACL injury prevention and postoperative recovery.

### ACL injury risk

Vitamin D is a crucial modulator of matrix metalloproteinase (MMP)‐9, exhibiting an inverse relationship with the inflammatory factor. An imbalance between matrix MMPs and their inhibitors, known as tissue inhibitors of metalloproteinases, has been linked to tendinopathies and ligament injuries. Moreover, vitamin D can activate p38 pathways and extracellular signal‐regulated kinases, inhibiting proinflammatory cytokines, including interleukin‐6 and tumour necrosis factor‐alpha [32].

A range of risk variables correlates with knee health post‐ACLR, including impairment of the knee extensor muscles. The notion that muscle weakness, particularly in the quadriceps, serves as a risk factor for osteoarthritis highlights the need to maintain or enhance quadriceps strength to safeguard against the harmful effects on cartilage and surrounding tissues that trigger and advance the disease. Prior research indicates that low levels of circulating 25(OH)D (i.e., 25(OH)D < 30 ng/mL or hypovitaminosis D) adversely affect the recovery of muscle strength in the affected limb during rehabilitation following ACL reconstruction [20, 38]. Reduced serum 25(OH)D levels are also correlated with additional risk factors for osteoarthritis, including primary ACL tears, revision ACLR, and quadriceps atrophy following ACLR. While serum 25(OH)D can be easily altered by various interventions in osteoarthritis (OA) and other disorders, it remains uncertain whether elevating serum 25(OH)D mitigates muscle weakness and other factors associated with OA, as well as prevents the onset of knee OA after ACLR.

The finding that vitamin D deficiency is associated with an increased risk of ACL injury, as reported by Albright et al., aligns with the broader literature linking vitamin D to musculoskeletal health [18, 27, 36]. Several studies have demonstrated that low serum 25‐hydroxyvitamin D levels can lead to weakened muscle and tendon structures, potentially increasing the risk of soft tissue injuries, including ACL tears. Vitamin D is known to enhance muscle function, and its deficiency may impair the neuromuscular control needed to prevent high‐risk movements during sports activities. The significant increase in ACL injury incidence among vitamin D‐deficient individuals in Albright's study is consistent with other research showing that athletes with insufficient vitamin D levels experience a higher frequency of sports‐related injuries. Furthermore, lifestyle factors such as limited outdoor activity, sedentary habits, and dietary insufficiencies, influence vitamin D levels. These factors highlight the importance of addressing vitamin D insufficiency in populations at risk of ACL injuries, both through nutritional interventions and lifestyle modifications aimed at improving overall musculoskeletal health.

### Postoperative muscle strength recovery

The vitamin D system may play a regenerative role in damaged skeletal muscle, as evidenced by increased cellular turnover and improved muscular function following subcutaneous infusion of vitamin D3 after crush injury in rats. Initiating vitamin D3 treatment immediately post‐injury enhances cell proliferation in the interstitium of damaged skeletal muscle while concurrently reducing necrotic cells, indicating increased activity of mononuclear cells that may contribute to muscle repair, including various immune cells, macrophages and fibrogenic cells.

The role of vitamin D in muscle recovery post‐ACL reconstruction has been increasingly recognized, as supported by the studies reviewed here. Barker et al. observed that patients with higher vitamin D levels demonstrated significantly greater improvements in muscle strength following surgery. These findings align with previous studies showing that vitamin D facilitates muscle regeneration and recovery after injury or surgery [1]. Wen et al. corroborated these results, reporting a clear association between low vitamin D levels and greater reductions in muscle fibre CSA. This suggests that vitamin D's role in muscle strength preservation may be mediated by its influence on muscle protein synthesis and neuromuscular function. Given the significant impairments in postoperative recovery seen in patients with vitamin D deficiency, routine monitoring of vitamin D levels before ACL surgery could be beneficial in promoting optimal recovery.

Interestingly, Qiu et al. extended these findings to neuromuscular function, particularly in the uninjured limb, suggesting that maintaining sufficient vitamin D levels could help prevent further injuries during rehabilitation [16, 20, 31]. This adds to the body of evidence indicating that vitamin D may be crucial for maintaining overall knee stability post‐reconstruction, potentially reducing the risk of contralateral ACL injuries.

### Functional outcomes

While vitamin D appears to influence muscle recovery, its impact on broader functional outcomes, such as return to sport and quality of life, remains unclear [6, 14, 37, 38]. Gupta et al. found no significant differences in key functional scores (Lysholm and WOMAC) across different vitamin D levels, although those with higher vitamin D levels did exhibit slightly better Tegner activity scores. This finding is in line with studies that have struggled to establish a clear connection between vitamin D levels and subjective functional outcomes following ACL reconstruction. It is possible that other factors, such as adherence to rehabilitation protocols, pre‐existing muscular strength or genetic factors, may play a more substantial role in determining long‐term functional outcomes than vitamin D levels alone. Nevertheless, the potential link between vitamin D and return‐to‐sport performance, as suggested by the improved Tegner scores in the Gupta study, warrants further investigation.

### Bone health and skeletal muscle loss

The relationship between vitamin D and bone health post‐ACL reconstruction remains inconclusive based on current literature [9, 17]. Wen et al. found no significant association between vitamin D levels and BMD loss, despite clear evidence of its role in preventing muscle loss [28, 33]. This is somewhat surprising, as vitamin D is well‐known for its role in calcium metabolism and bone health. Other studies have demonstrated that vitamin D deficiency is associated with reduced BMD, particularly in the elderly or in individuals undergoing orthopaedic procedures. However, in the context of ACL reconstruction, it may be that the timeframe for observing changes in bone density is longer than the typical follow‐up periods in the studies reviewed. Future research should aim to investigate the long‐term effects of vitamin D on bone health after ACL surgery, particularly over follow‐up periods exceeding one year.

### Animal studies

In addition to human studies, animal models have been employed to explore the relationship between vitamin D and ligament health.

Boyan et al. showed in a rat model that 24R,25‐dihydroxyvitamin D3 supplementation protected against articular cartilage damage following ACL transection, emphasizing the role of vitamin D in mitigating joint degeneration [7].

Clements et al. looked at vitamin D levels in dogs post‐ACL surgery and found no significant difference between healthy dogs and those with cranial cruciate ligament rupture (CCLR) [10].

A subsequent study by Clements further examined the role of vitamin D in clinical outcomes after CCLR surgery in dogs, but found no significant correlation between preoperative vitamin D levels and postoperative recovery, suggesting that the role of vitamin D in ACL injury recovery may vary between species and contexts [11].

These findings underscore the complexity of vitamin D's role in ligament health and recovery. While vitamin D appears beneficial in mitigating cartilage damage in rats, its effect on postoperative outcomes in dogs remains inconclusive. Further research is needed to bridge the gap between animal and human studies and to clarify the role of vitamin D in ligament repair and recovery.

### Strengths and limitations

A major strength of our systematic review is its focused examination of the relationship between vitamin D levels and outcomes related to ACL injuries and reconstruction. By including a range of observational studies, we provide a thorough overview of the available evidence, identifying vitamin D as a potential modifiable factor in ACL injury risk and postoperative recovery. Additionally, the strict inclusion and exclusion criteria ensured that only studies with relevant endpoints and adequate data quality were included. This allows for more accurate conclusions regarding the role of vitamin D in musculoskeletal health, specifically concerning ACL injuries.

However, several limitations must be acknowledged. The most significant limitation is the lack of RCTs which are considered the gold standard for establishing causal relationships. As our review is based solely on observational studies (cohort and cross‐sectional designs), the findings may be more prone to biases such as confounding and selection bias. Moreover, the heterogeneity of the included studies, particularly in terms of vitamin D dosing, assessment of outcomes, and follow‐up duration, makes it difficult to synthesize the results into a unified conclusion.

This issue is significant, as it undermines the validity and utility of reviews employing narrative synthesis, hence heightening the potential of contributing to research waste. Narrative synthesis is aggregating research findings into a cohesive written account, detailing variations in study parameters such as context and validity, and frequently employing tables and graphs to present results [8].

Many of the studies also had relatively small sample sizes, which may limit the generalizability of the results to broader populations. Finally, long‐term outcomes, especially related to bone health and functional recovery, were not consistently evaluated across the studies, leaving a gap in understanding the full impact of vitamin D on recovery post‐ACL reconstruction.

## CONCLUSION

This systematic review suggests that low vitamin D levels increase the risk of ACL injuries and hinder postoperative muscle recovery. Adequate vitamin D levels are linked to better muscle strength after ACL reconstruction, although the impact on broader functional outcomes like return to sport is not clear. Further research is needed to establish clear guidelines on vitamin D's role in long‐term recovery and injury prevention.

## AUTHOR CONTRIBUTIONS

**Andrea Pasquini**: Conceptualization; visualization; writing—original draft preparation; writing—review and editing. **Radu Prejbeanu**: Conceptualization; writing—review and editing; supervision. **Octav Russu**: Conceptualization; writing—review and editing. **Riccardo D'Ambrosi**: Conceptualization; visualization; writing—original draft preparation; writing—review and editing; supervision. **Cosmin Ioan Faur**: Conceptualization; writing—review and editing. **Giulio Maria Marcheggiani Muccioli**: Conceptualization; writing—original draft preparation; writing—review and editing; supervision. **Mihail Lazar Mioc**: Conceptualization; writing—original draft preparation; writing—review and editing. All authors have read and agreed to the published version of the manuscript.

## CONFLICT OF INTEREST STATEMENT

The authors declare no conflicts of interest.

## ETHICS STATEMENT

The ethics statement is not available.

## Supporting information

Supporting information.

## Data Availability

Data are available on request from the corresponding author.
