# Supplementary material for: Effect of vitamin D on anterior cruciate ligament injury rates and post‐reconstruction function—A systematic review
Source: J Exp Orthop. 2025 Apr 1;12(2):e70224. doi: 10.1002/jeo2.70224 (PMC11959628; doi:10.1002/jeo2.70224)
Supplement: Supplementary file 1 — Supporting information. [file JEO2-12-e70224-s001.docx]

**Supplementary Appendix 1**

**COCHRANE LIBRARY**

ID Search Results

#1 ("anterior cruciate ligament reconstruction"):ti,ab,kw 2295

#2 ("vitamin D"):ti,ab,kw 15209

#3 #1 AND #2 6

6 results

**EMBASE**

(Vitamin D) AND (Anterior Cruciate Ligament Injuries OR Anterior Cruciate Ligament Reconstruction)

76 results

**PUBMED**

("Vitamin D"[Mesh] OR "Vitamin D Deficiency"[Mesh] OR "Vitamin D" OR “25-hydroxyvitamin D“ OR “25(OH)D“ OR “serum vitamin D” OR “Vitamin D Deficienc*” OR “Serum 25-hydroxyvitamin D level*” OR “Low vitamin D” OR “Hypovitaminosis D”)

AND

("Anterior Cruciate Ligament Injuries"[Mesh] OR "Anterior Cruciate Ligament Reconstruction"[Mesh] OR "Anterior Cruciate Ligament Injuries"[Mesh] OR "ACL" OR "acl ruptur*" OR “ACL Injur*” OR “Anterior Cruciate Ligament Injur*” OR “Anterior Cruciate Ligament Tear*” OR “ACL Tear*” OR "Anterior cruciate ligament" OR “Anterior cruciate ligament reconstruction” OR “ACLR” OR "Anterior Cruciate Ligament Surgery")

32 results

31 after language filter ENG

**SCOPUS**

vitamin AND d AND acl AND reconstruction AND PUBYEAR > 2013 AND PUBYEAR < 2025 AND ( LIMIT-TO ( LANGUAGE , "English" ) OR EXCLUDE ( LANGUAGE , "French" ) OR EXCLUDE ( LANGUAGE , "Portuguese" ) ) AND ( LIMIT-TO ( SUBJAREA , "MEDI" ) ) AND ( EXCLUDE ( DOCTYPE , "bk" ) OR EXCLUDE ( DOCTYPE , "ch" ) OR EXCLUDE ( DOCTYPE , "cp" ) OR EXCLUDE ( DOCTYPE , "no" ) OR EXCLUDE ( DOCTYPE , "ed" ) )

190 results

**WEB of SCIENCE**

((ALL=(acl injury)) OR ALL=(acl reconstruction)) AND ALL=(vitamin d)

13 results
